# Characterisation of Ventricular Nucleotide Metabolism and Clinical Predictors Associated with the Onset of Atrial Fibrillation Following Cardiac Surgery

**DOI:** 10.3390/jcm14134777

**Published:** 2025-07-07

**Authors:** Daniel Paul Fudulu, Arnaldo Dimagli, Marco Moscarelli, Rahul Kota, Tim Dong, Marco Gemelli, Manraj Sandhu, Saadeh Suleiman, Gianni D. Angelini

**Affiliations:** 1 Bristol Heart Institute, University of Bristol, Upper Maudlin St., Bristol BS2 8HW, UK; arnaldo.dimagli@bristol.ac.uk (A.D.); qd18830@bristol.ac.uk (T.D.); marco.gemelli.01@gmail.com (M.G.); manraj.sandhu@bristol.ac.uk (M.S.); g.d.angelini@bristol.ac.uk (G.D.A.); 2 GVM Care & Research, Department of Cardiovascular Surgery, Anthea Hospital, 70124 Bari, Italy; m.moscarelli@imperial.ac.uk; 3 Bristol Medical School, University of Bristol, Bristol BS8 1TH, UK; rkota52@gmail.com (R.K.); m.s.suleiman@bristol.ac.uk (S.S.)

**Keywords:** atrial fibrillation, nucleotides, nucleotide metabolism, cardiac surgery

## Abstract

**Introduction:** Postoperative atrial fibrillation (POAF) is a common complication after heart surgery, adversely impacting clinical outcomes and healthcare costs. Little is known about the dynamics of nucleotide metabolism associated with the development of POAF at a ventricular level. We conducted a post hoc trial analysis to investigate the changes in ventricular adenine nucleotides and the clinical predictors associated with the development of AF. **Methods:** Using data from a randomised trial, we analysed ATP/ADP, ATP/AMP, and energy charges in left and right ventricular biopsies of patients who developed AF compared to non-AF patients. A logistic regression model was used to understand the predictors associated with the development of atrial fibrillation in this cohort. **Results:** We analysed adenine nucleotide levels available in 88 patients who underwent coronary artery bypass grafting (CABG) (*n* = 65) and aortic valve replacement (AVR) (*n* = 23), out of which 27 (31%) developed a new onset of AF. Seventeen (43.4%) patients in the CABG group and ten (26.15%) in the AVR group developed AF. The patients who developed postoperative AF had longer cross-clamp times for CABG (*p* = 0.013) and AVR (*p* = 0.002). The most significant predictors for AF development were age (*p* = 0.003) and cross-clamp time (*p* = 0.012). In patients undergoing CABG who developed AF, we found a significant drop in post-reperfusion ATP/ADP and ATP/AMP ratios compared to pre-reperfusion. This was not significant for the patients who underwent AVR. Furthermore, the patients who underwent CABG and developed AF had higher pre- and post-reperfusion ATP/ADP ratios and energy charges than non-AF patients, suggesting a higher reserve of cardiac nucleotides. **Conclusions:** The development of postoperative atrial fibrillation is associated with intraoperative changes in the ventricular adenine nucleotide metabolism of patients undergoing CABG. In the clinical analysis, age and cross-clamp time were significant predictors of AF development.

## 1. Introduction

Postoperative atrial fibrillation (PAF) is the most common arrhythmic complication following cardiac surgery. It is a highly relevant clinical complication, resulting in excess mortality, morbidity, and hospital costs [1,2,3]. Extensive research has focused on understanding the pathogenesis of PAF at a tissue level and the clinical risk factors predictive of PAF [4]. Alterations in the adenine nucleotide metabolism of the cardiac muscle have been studied in the atrial tissue in the context of chronic atrial fibrillation or acute stretch-related AF [5,6,7,8,9]. However, no studies have investigated adenine nucleotide metabolism at a ventricular level preceding the development of atrial fibrillation after cardiac surgery. The clinical risk factors predictive of new-onset atrial fibrillation after cardiac surgery have been extensively studied, and they are related to both preoperative characteristics and intraoperative factors [10]. The primary aim of our work was to investigate whether changes in high-energy phosphate metabolism at the ventricular level are associated with the development of atrial fibrillation after cardiac surgery and their correlation with clinical characteristics. We therefore hypothesised that the development of postoperative atrial fibrillation is associated with intraoperative changes in adenine nucleotide metabolism at a ventricular level. As a secondary objective, we examined the clinical predictors of atrial fibrillation related to PAF development. To test the above hypothesis, we conducted a post hoc, retrospective analysis of the RIPC trial database [11]. 

## 2. Methods

The RIPC (remote ischaemic preconditioning) trial aimed to assess the effect of remote ischaemic preconditioning on cardiac injury, metabolic stress, and inflammatory response in 124 patients undergoing isolated coronary artery bypass grafting (CABG) and aortic valve replacement (AVR) [11]. One of the trial’s endpoints was the analysis of adenine nucleotides in the left and right ventricular myocardial biopsies, obtained using Trucut myocardial biopsies pre- and post-reperfusion. Of note, in the RIPC trial, there was no effect of the remote ischaemic preconditioning intervention compared to the sham. Therefore, we have included both arms of the study in the post hoc analysis (sham and intervention) and performed the analysis. Furthermore, the RIPC trial intervention was equally distributed between AF and non-AF cohorts. We analysed the ATP, ADP, and AMP changes in the ventricular biopsies available for 88 patients measured pre- and post-op (i.e., 176 time points in total). The ventricular nucleotides were measured using high-performance liquid chromatography (HPLC), as previously described in the original paper [11] (Moscarelli et al., 2019) [11]. Energy charge was calculated as follows: energy charge = (ATP + 0.5 × ADP)/(ATP + ADP +AMP) [12]. For the nucleotide analysis, we used GraphPad Prism version 8.4.3 GraphPad Software, La Jolla, CA, USA, www.graphpad.com (accessed on 11 May 2025). Because some nucleotide measurements were missing per time point data (6 timepoints, 3.4%), we performed the analysis by fitting a mixed model to assess changes in nucleotide metabolism between AF and non-AF patients. Missing data were handled by exclusion. We used Sidak’s multiple comparisons test with correction across multiple endpoints to assess changes between pre- and post-reperfusion nucleotide metabolism within the AF and non-AF patients. The AF endpoint was defined as any new episode of postoperative atrial fibrillation detected on ECG monitoring during admission by the medical team.

We used R version 1.4.1717, gtsummary, sJPlot, glm and MASS packages to analyse clinical predictors for AF. For categorical variables analysis, we used the chi-square test (with continuity correction), and for continuous variables, a t-test. Categorical variables were summarised as counts and percentages. Continuous variables were summarised as mean and standard deviation (SD) or median and interquartile range. The Shapiro–Wilk test was used to assess the normality of the distribution of continuous data. Non-normally distributed data were averaged as a median with interquartile range (IQR) and analysed using a rank-sum test. All the analysed data were anonymised; hence, there was no need to obtain ethics approval in addition to the original ethics approval for this post hoc analysis.

We used a generalised linear model (logistic regression) to assess the effect of the following predictors on the development of postoperative atrial fibrillation: operation type (AVR or CABG), age, gender, BMI, LV function, diabetes, preoperative creatinine, smoker status, peripheral vascular disease, pulmonary disease, previous myocardial infarction, hypertension, preoperative beta-blocker use, and cross-clamp time. Three patients in the original trial had preoperative AF and were excluded from the analyses. 

## 3. Results

### 3.1. Baseline Characteristics

Both AVR and CABG patients who developed AF had significantly longer cross-clamp times than non-AF patients (Table 1). In addition, the CABG patients were significantly older (72 vs. 64, *p* = 0.02). We found no significant differences in the rest of the baseline characteristics between patients with or without AF (Table 1).

### 3.2. Unadjusted Clinical Outcomes 

The AF patients had more extended hospital stays (*p* < 0.002). There was no stroke or mortality in either group. 

### 3.3. Clinical Predictors of AF

In the logistic regression model, the most significant predictors for PAF were age (*p* = 0.002) and cross-clamp time (*p* = 0.019) (Table 2).

### 3.4. Changes in Ventricular Nucleotide Metabolism 

We analysed ventricular adenine nucleotide levels in 88 patients undergoing available biopsies who underwent CABG (*n* = 65) and AVR (*n* = 23). Seventeen (43.4%) patients in the CABG group and ten patients (26.15%) in the AVR group developed AF. 

### 3.5. CABG Group

**Figure 1:**
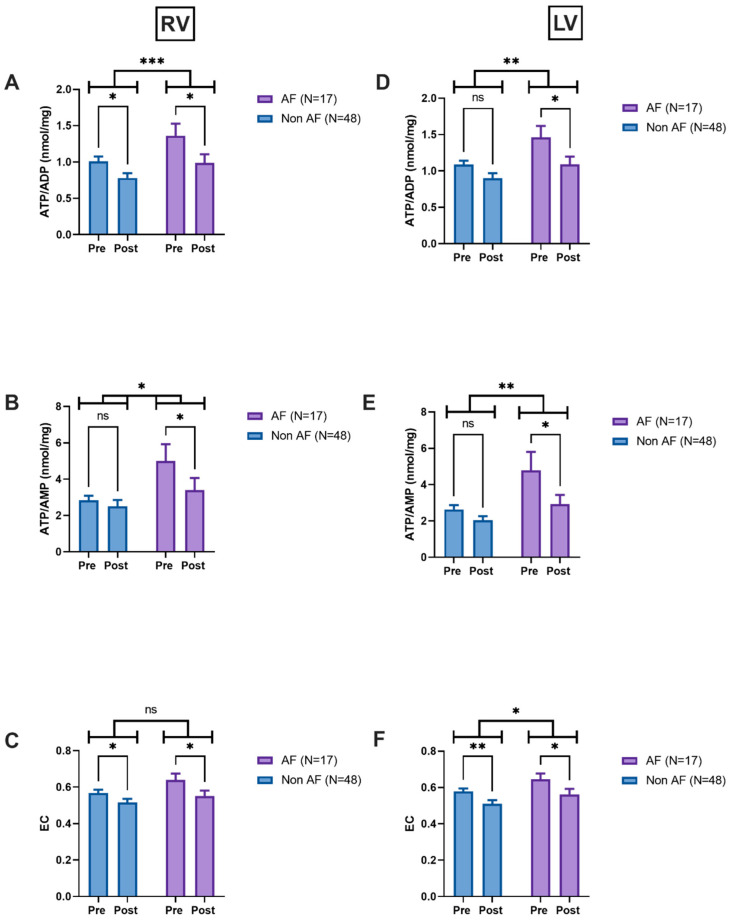
Changes in nucleotide metabolism for CABG patients. (**A**) Changes in ATP/ADP ratios in the right ventricle. (**B**) Changes in ATP/AMP ratios in the right ventricle. (**C**) Changes in EC in the right ventricle. (**D**) Changes in ATP/ADP ratios in the left ventricle. (**E**) Changes in ATP/AMP ratios in the left ventricle. (**F**) Changes in EC in the left ventricle. Data are mean ± SEM; data were analysed using a mixed model followed by Sidak’s multiple comparison test. * *p* < 0.05; ** *p* < 0.01; *** *p* < 0.001 vs. non-AF group. Abbreviations: RV—right ventricle; LV—left ventricle. Adenosine triphosphate (ATP); Adenosine diphosphate (ADP); Adenosine monophosphate (AMP). EC—energy charge, Pre—pre-reperfusion, Post—post-reperfusion.

In the right ventricular (RV) samples, AF/non-AF status had a significant effect on ATP/ADP (*p* = 0.013). The post-reperfusion ATP/ADP ratios were significantly lower compared to pre-reperfusion ratios in AF (*p* = 0.016) and non-AF groups (*p* = 0.03), respectively (Figure 1A). Similarly, we found a significant effect of AF/non-AF status on ATP/AMP ratios (*p* = 0.009). In the post hoc analysis, the ATP/AMP ratios were lower post-reperfusion compared to pre-reperfusion in AF samples only (*p* = 0.035) (Figure 1B). There was no overall effect of AF versus non-AF status on energy charges (*p* = 0.08), but there was a significant drop in the energy charge post-reperfusion compared to pre-reperfusion levels in both groups, AF (*p* = 0.025) and non-AF (*p* = 0.042) (Figure 1C).

We noted similar patterns in the left ventricular (LV) biopsies. We found a significant effect of AF status versus non-AF on ATP/ADP ratios (*p* = 0.005). The ATP/ADP ratios were significantly lower (*p* = 0.012) in post-reperfusion samples versus pre-reperfusion in AF patients only (Figure 1D). In the AF patients, the ATP/AMP ratios significantly dropped post-reperfusion compared to pre-reperfusion (*p* = 0.02) (Figure 1E). The energy charges dropped post-reperfusion versus pre-reperfusion for both AF (*p* = 0.043) and non-AF patients (*p* = 0.005) (Figure 1F). Finally, the ATP/ADP ratio, ATP/AMP ratios and energy charges (pre-and post-reperfusion) were significantly higher in AF versus non-AF patients (Figure 1A–F), except for energy charges in the RV, where we found no significant difference (Figure 1C).

### 3.6. AVR

**Figure 2:**
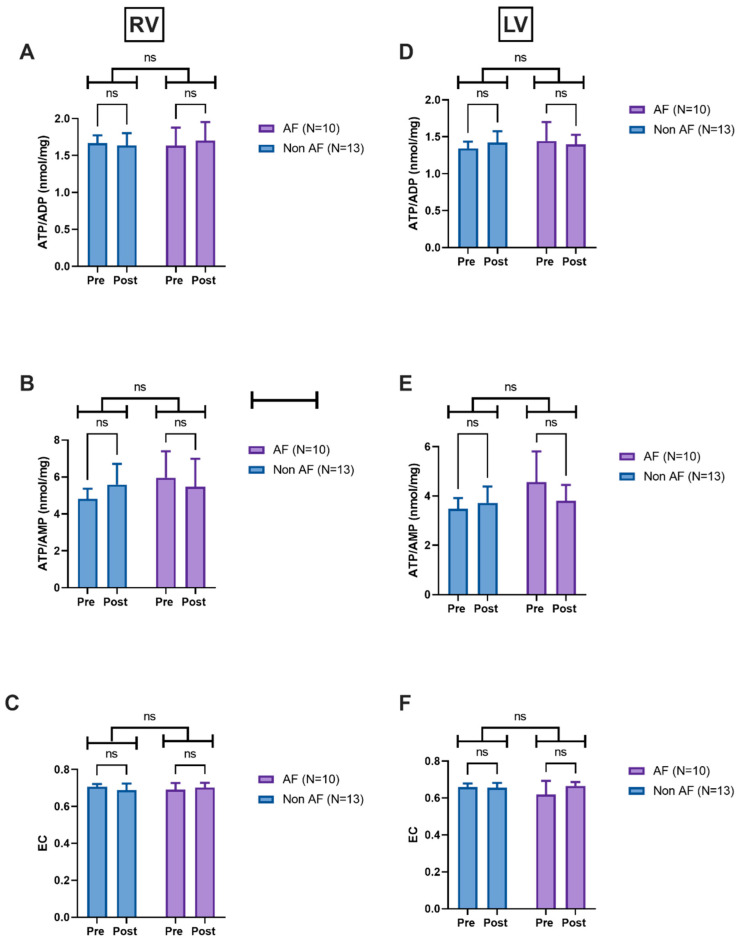
Changes in nucleotide metabolism for AVR patients. (**A**) Changes in ATP/ADP ratios in the right ventricle. (**B**) Changes in ATP/AMP ratios in the right ventricle. (**C**) Changes in EC in the right ventricle. (**D**) Changes in ATP/ADP ratios in the left ventricle. (**E**) Changes in ATP/AMP ratios in the left ventricle. (**F**) Changes in EC in the left ventricle. Data are mean ± SEM; data were analysed using a mixed model followed by Sidak’s multiple comparison test. Abbreviations: RV—right ventricle; LV—left ventricle; ATP—adenosine triphosphate; ADP—adenosine diphosphate; AMP—adenosine monophosphate; EC—energy charge, Pre—pre-reperfusion, Post—post-reperfusion.

We found no significant differences in ATP/ADP, ATP/AMP, or energy charge between AF and non-AF patients undergoing aortic valve replacement (Figure 2A–F).

## 4. Discussion

In this post hoc trial analysis, we investigated the ventricular-level changes in nucleotide metabolism that precede the development of new-onset postoperative atrial fibrillation. To our knowledge, this is the first study reporting such findings. Adenosine triphosphate (ATP) and the energy released from its hydrolysis to ADP and AMP are the primary energy sources for cardiac work and the maintenance of excitability [13]. Therefore, changes in atrial bioenergetics due to mitochondrial dysfunction could be associated with increased arrhythmogenicity and the development of AF [8,14]. While the pathogenesis of postoperative atrial fibrillation is multifactorial, data suggest a correlation between atrial ischaemia and the duration of aortic cross-clamp [4]. Furthermore, it is well established that myocardial hypoperfusion is related to reduced levels of high-energy phosphates such as ATP and to no recovery if the aortic cross-clamp time is too long [15].

Firstly, we noted significantly higher ATP/ADP and ADP/AMP ratios and energy charges (pre-and post-reperfusion) in the CABG patients that developed AF. Paradoxically, this suggests that the hearts of patients developing AF have a higher reserve of cardiac nucleotides. These changes were not present in AVR patients. 

In the CABG patients that developed AF, we also noted a significant drop in ATP/ADP and ATP/AMP ratios post-reperfusion compared to non-AF, except in the right ventricle, where ATP/ADP ratios dropped post-reperfusion in both AF and non-AF patients. The ATP/ADP and ATP/AMP ratios dropped post-reperfusion in AF patients, suggesting that the mechanism related to AF development could be related to intraoperative ischaemia. This correlates with AF patients’ longer aortic cross-clamp times compared to non-AF patients. Furthermore, cross-clamp time was an independent predictor for AF development in our analysis. These findings argue for rigorous heart protection and the minimisation of cross-clamp time to prevent postoperative AF development. 

An important finding is that we noted no changes in ventricular nucleotides associated with AF development in patients undergoing AVR. This could be likely explained by the different metabolic demands of the hypertrophic myocardium of AVR patients, which has been shown to have a higher concentration of ventricular nucleotides compared to patients with ischaemic heart disease who undergo CABG [12]. This could explain a higher tolerance to ischaemia. Furthermore, patients with ischaemic heart disease have high-grade coronary stenoses that could result in inadequate cardioprotection during cardioplegia administration. 

These findings also highlight that the dynamics in nucleotide metabolism at the ventricular level could be related to the development of atrial fibrillation. We had no atrial samples to assess if these changes in nucleotide metabolism at a ventricular level are also mirrored at an atrial level. Nevertheless, one study by Tsuboi et al. found that mitochondrial DNA deletions in the human atrium obtained during cardiac surgery were associated with a reduction in adenine nucleotides and were more prevalent in patients with AF [9]. Finally, the patients who developed AF were older and more prone to developing AF. This is a well-established risk factor we found in our clinical analysis. Furthermore, high-energy phosphate metabolism changes with ageing; hence, some of the changes we noted in the CABG patients could be related to the ageing process [16]. 

The strength of our work is the availability of ventricular muscle biopsies, which allowed us to assess ventricular nucleotide metabolism changes that precede AF development and to correlate them with clinical prediction data from a trial-quality database. To our knowledge, this is the first study reporting such findings. However, we must acknowledge that this post hoc trial analysis should be viewed as a hypothesis-generating study only. We need to take into account the relatively small sample size of the study, which was not statistically powered for the endpoints we investigated. These considerations limit the generalizability of the results and suggest the need for further studies. Moreover, we found no changes in nucleotide metabolism among patients undergoing AVR, which could be due to the limited sample size of this procedural subgroup. Finally, issues such as selection bias or unmeasured confounding cannot be accounted for because of the lack of randomisation according to AF status. 

In conclusion, we demonstrate that the development of postoperative atrial fibrillation after CABG is associated with significant alterations in pre- and post-reperfusion ATP/ADP and ATP/AMP ratios, likely related to intraoperative ischaemia.

## Figures and Tables

**Table 1:** Baseline characteristics.

	CABG (N = 65)			AVR (N = 23)		
Characteristic	No AF, N = 48 ^1^	AF, N = 17 ^1^	*p*-Value ^2^	No AF, N = 13 ^1^	AF, N = 10 ^1^	*p*-Value ^2^
Age (years)	64 (59, 68)	72 (66, 76)	0.002	65 (61, 76)	74 (70, 77)	0.3
Male gender	39 (81%)	11 (65%)	0.2	11 (85%)	8 (80%)	>0.9
Body mass index (kg/m^2^)	29.2 (25.4, 32.4)	27.0 (25.1, 28.8)	0.2	27 (24, 34)	27 (26, 33)	0.9
LV function			0.7			>0.9
LV good > 50%	39 (81%)	13 (76%)		12 (92%)	10 (100%)	
LV moderate < 50% and >30%	9 (19%)	4 (24%)		1 (7.7%)	0 (0%)	
Creatinine (mg/dL)	79 (69, 99)	83 (70, 90)	0.8	92 (80, 98)	88 (66, 96)	0.5
CVA/TIA (%)	5 (10%)	0 (0%)	0.3	2 (15%)	1 (10%)	>0.9
Smoking status			0.7			0.7
Ex-smoking	7 (15%)	1 (5.9%)		4 (31%)	4 (40%)	
Smoking	20 (42%)	9 (53%)		9 (69%)	6 (60%)	
Extracardiac arteriopathy	1 (2.1%)	0 (0%)	>0.9	0 (0%)	1 (10%)	0.4
Pulmonary disease	8 (17%)	3 (18%)	>0.9	2 (15%)	3 (30%)	0.6
Neurological dysfunction	0 (0%)	0 (0%)		1 (7.7%)	0 (0%)	>0.9
Previous MI	8 (17%)	2 (12%)	>0.9	1 (7.7%)	1 (10%)	>0.9
Hypertension	41 (85%)	14 (82%)	0.7	10 (77%)	9 (90%)	0.6
Hypercholesterolaemia	41 (85%)	12 (71%)	0.3	10 (77%)	5 (50%)	0.2
Diabetes			>0.9			0.13
NIDDM	10 (21%)	3 (18%)		0 (0%)	0 (0%)	
IDDM	4 (8.3%)	1 (5.9%)		1 (7.7%)	4 (40%)	
Pre-op betablocker	30 (62%)	10 (59%)	0.8	5 (38%)	5 (50%)	0.7
Cardiopulmonary bypass time (min)	80 (72, 92)	80 (75, 99)	0.6	83 (62, 98)	96 (89, 114)	0.051
Aortic cross clamp time (min)	42 (37, 50)	49 (44, 60)	0.013	55 (48, 65)	68 (62, 83)	0.028

^1^ Median (IQR); *n* (%); ^2^ Wilcoxon rank sum test; Fisher’s exact test; Wilcoxon rank sum exact test. AF: atrial fibrillation; AVR: aortic valve replacement; CABG: coronary artery bypass grafting; CVA: cerebral vascular accident; IDDM: insulin-dependent diabetes mellitus; LV: left ventricular function; MI: myocardial infarction; NIDDM: non-insulin-dependent diabetes mellitus; TIA: transient ischaemic attack.

**Table 2:** Predictor variables for new onset of postoperative atrial fibrillation.

Characteristic	Log(OR) ^1^	95% CI ^1^	*p*-Value
Operation type (AVR/CABG)	0.64	−1.0, 2.4	0.5
Sex	−0.24	−2.2, 1.6	0.8
Age (years)	0.14	0.06, 0.24	0.003
Body mass index (kg/m^2^)	−0.01	−0.12, 0.10	0.9
LV function	−0.19	−2.3, 1.8	0.9
Diabetes	0.35	−0.36, 1.1	0.3
Pre-op creatinine	−0.02	−0.07, 0.02	0.3
Pre-op CVA/TIA	−3.6	−8.8, −0.25	0.091
Smoker	0.34	−0.33, 1.1	0.3
Peripheral vascular disease	2.1	−2.5, 6.4	0.3
Pulmonary disease	0.07	−1.7, 1.8	>0.9
Previous MI	0.44	−1.5, 2.3	0.6
Hypertension	−0.28	−2.1, 1.6	0.8
Hypercholesterolaemia	−1.3	−2.9, 0.28	0.12
Pre-op betablocker	0.67	−0.68, 2.2	0.3
Aortic cross clamp time (min)	0.05	0.01, 0.10	0.012

^1^ OR = Odds Ratio; CI = Confidence Interval.

## Data Availability

Original contributions presented in the study are included in the article. Further inquiries can be directed to the corresponding author.
